# Nocardia Rubra Cell Wall Skeleton Up-Regulates T Cell Subsets and Inhibits PD-1/PD-L1 Pathway to Promote Local Immune Status of Patients With High-Risk Human Papillomavirus Infection and Cervical Intraepithelial Neoplasia

**DOI:** 10.3389/fimmu.2020.612547

**Published:** 2021-01-20

**Authors:** Wei Chen, Yi Zhang, Chunfang Zhao, Suxia Shao, Yanan Zhang, Xuehui Li, Xue Bai, Qianyu Guo, Qianwen Liu, Junmin Tang, Lei Zhang

**Affiliations:** ^1^ Department of Histology and Embryology, Hebei Medical University, Shijiazhuang, China; ^2^ Department of Medicine, Weihai Greatest Pharmaceutical Research Institute Co., Ltd., Weihai, China; ^3^ Department of Gynaecology, The First Hospital of Hebei Medical University, Shijiazhuang, China; ^4^ Department of Gynaecology, The Fourth Hospital of Hebei Medical University, Shijiazhuang, China; ^5^ Department of Histology and Embryology, Peking University Health Science Centre, Beijing, China

**Keywords:** Nocardia rubra cell wall skeleton, high-risk human papillomavirus, cervical intraepithelial neoplasia, CD4^+^ T cell, CD8^+^ T cell, programmed cell death-1, programmed cell death-ligand 1

## Abstract

The Nocardia rubra cell wall skeleton (Nr-CWS) for external use is an immune enhancer, which has been widely used in human cervix diseases such as cervical erosion, but the mechanism of Nr-CWS enhancing immunity is still unclear. The purpose of this study was to explore the effect and mechanism of Nr-CWS on the local immune status of cervical tissue in patients with high-risk human papillomavirus (HR-HPV) infection and cervical precancerous lesion, cervical intraepithelial neoplasia (CIN). The recruited patients with HR-HPV infection and CIN were treated with Nr-CWS. The specimens were taken from these patients before and after local application of Nr-CWS respectively. The normal control specimens were tested simultaneously. Serial section analysis of immunohistochemistry and co-expression analysis were performed to characterize populations of T cells and the expressions of programmed cell death-1 (PD-1) and programmed cell death-ligand 1 (PD-L1). The levels of cytokines in local cervical tissue were also detected. Nr-CWS significantly increased T cells including CD4^+^, CD8^+^ T cells, and reduced the expression of PD-L1 in the patients’ local cervical tissues. Co-expression analyses showed that the proportions of PD-1^+^CD4^+^ cells in CD4^+^ T cells and PD-1^+^CD8^+^ cells in CD8^+^ T cells decreased after Nr-CWS application. Furthermore, the increase in the number of immune cells was accompanied by increased pro-inflammatory cytokines interleukin-12 (IL-12), interferon-γ (IFN-γ), tumor necrosis factor-α (TNF-α), and decreased suppressive cytokine IL-10. The results indicate that Nr-CWS, as an immunotherapeutic agent for HR-HPV infection and CIN, plays an immune promoting role related to the upregulation of T cell subsets and the inhibition of PD-1/PD-L1 pathway.

## Introduction

Cervical cancer is the fourth most common cancer in females worldwide. China together with India, contributed more than one-third of the global cervical burden in 2018, with 106,000 cases and 48,000 deaths in China (1). Cervical cancer is still an important cause of mortality among females in developing countries (2). It has been well known that human papillomavirus (HPVs) are the etiological agents of cervical cancer and its premalignant precursor cervical intraepithelial neoplasia (CIN) (3). However, not all women who harbor HPV infection will develop cervical cancer. With normal immune function, most HPV infections can be cleared by an incompletely understood immune response within 6–18 months (4). Nevertheless, the risk of CIN increases with the type of HPV, duration of infection, immunosuppression, and environmental factors like cigarette smoking in the patient. The presence of persistent infection with high-risk HPV (HR-HPV) (5) and those who have associated cofactors like immunodeficiency or smoking are at higher risk for the progression of lesions to the development of invasive cancer. In China, the reported HR-HPV persistent infection rate among women (ages 16–69) was 13.30%–22.94% (6). It was reported that 21% of patients progressed to CIN 2 or higher, if HR-HPV infection persisted for more than 1 year (7).

The host immune response serves a pivotal role in eliminating HR-HPV and determining the regression of a cervical abnormality or persistence and progression to a malignancy (8). Improving body’s immunity to HR-HPV and avoiding immune escape should be important methods for the treatment of CIN and even cervical cancer.

The Nocardia rubra cell wall skeleton (Nr-CWS) is the cell wall skeleton obtained from N. rubra, a gram-positive bacterium, and mainly contains arabinogalactan, muramic acid and mucopeptide. Nr-CWS was first reported by Azuma et al. in 1976 (9) and was subsequently shown to exert antitumor effects and affect the complex immune network formed by interactions of immunocompetent cells (10). Recently, it has been proved *in vitro* that Nr-CWS could stimulate macrophages (11), dendritic cells, natural killer cells (12), CD4^+^ T cells (13), and CD8^+^ T cells (14), which imply its potential application in immunotherapy.

As an approved National Category II New Drug in China, the Nr-CWS for external use has been used for human cervix diseases, but the mechanism of Nr-CWS enhancing immunity in cervix is still unclear. The purpose of this study was to explore the effect and mechanism of Nr-CWS on the local immune status of cervical tissue in patients with HR-HPV infection and CIN, revealing its potential broad application prospects in the immunotherapy of cervical precancerous lesions and even cancer.

## Materials and Methods

### Reagents

The Nr-CWS for external use (Drug approval number: S20030009) was provided by Weihai Greatest Pharmaceutical Research Institute Co., Ltd. (China).

### Samples and Patients

After obtaining ethical approval from the First Hospital and the Fourth Hospital of Hebei Medical University, People’s Republic of China, the study participants were recruited from these two hospitals in 2019 and 2020. All participants gave written informed consent. Available clinical information included the Pap smear report, histology diagnosis, HPV DNA genotyping, prior cervical pathology, and HPV history.

Twenty-three patients suffered with CIN and HR-HPV infection in female lower genital tract were recruited and treated with Nr-CWS. HPV DNA genotyping and biopsy report were the gold standards for defining final disease status. Participants with the following conditions were excluded from the study: pregnancy, women with any signs and symptoms of sexually transmitted infections, severe heart, lung, liver and kidney disease, any other neoplastic diseases, immunocompromised state, and those under any anti-inflammatory or immunosuppressive treatment. The cervical specimens were taken from these patients at the times before and 1 and 3 months after local application of Nr-CWS respectively. During the re-examination 3 months after Nr-CWS treatment, HPV DNA genotyping and biopsy were detected.

Five normal control cervical specimens were taken from the patients without HPV infection who underwent hysteroscopic endometrial electrotomy or hysteroscopic transcervical resection of myoma because of their endometrium polyp or submucosal myoma of uterus.

### Nr-CWS Administration Method

The patients were given Nr-CWS on the 3^rd^ day after menstruation. The drug was administered once every other day at a dose of 120 μg for 10 times. The cervix was exposed and cleaned with a cotton ball soaked with normal saline, and wiped repeatedly with a dry gauze to form minor wounds. The Nr-CWS solution (60 μg Nr-CWS dissolved in 0.5 ml normal saline) was injected into the cervical canal with a Pasteur pipette. The Nr-CWS solution (60 μg Nr-CWS dissolved in 2 ml normal saline) was soaked with a cotton ball with tail thread and then pressed on the cervical surface with forceps for 1 min. After Nr-CWS application, the patient can move freely after lying down for 10 min. The cotton ball with tail thread was kept in the body for 24 h and then taken out.

### Immunohistochemical Method

Immunohistochemical method was performed to detect the infiltration of T cells and the expressions of programmed cell death-1 (PD-1) and programmed cell death-ligand 1 (PD-L1) in the local cervix. Cervical tissue samples were fixed in 10% formalin. Immunohistochemistry was performed using our previously published protocol (15). In brief, tissue sections were incubated with one of the following primary antibodies: anti-CD3 (1 : 500; ZsBio, Beijing, China), anti-CD4 (1 : 500; Abways Technology, Shanghai, China), anti-CD8 (1 : 500; Abways Technology), anti-PD-1 (1 : 1000; Abcam, Cambridge, Massachusetts, USA) and anti-PD-L1 (1 : 1000; Abcam). Immunodetection was performed using an appropriate biotinylated immunoglobulin and a horseradish peroxidase-labeled avidin kit (ZsBio) with diaminobenzidine (DAB) as the substrate. Finally, the sections were lightly counterstained with hematoxylin for 30 s.

### Scoring System and Analysis of Immunohistochemical Method

The modified McCarty’s “H” scoring system was used to evaluate the immunohistochemistry method (16).

Modified H-score (0-300)=intensity (0-3) × percentage of immunohistochemical positive cells

One hundred cells were counted sequentially and percentage of immunohistochemical positive cells was calculated. In order to make intensity assessments more objective, the intensity of immunohistochemical positive was not assessed visually, but measured as mean grey value (MGV). The MGV was analysed using Image-J software (NIH, Bethesda, MA, USA). The following MGV ranges were used to grade the intensity of staining (191.5–255, 0 = negative; 127.5–191.5, 1 = weak positive; 63.5–127.5, 2 = moderate intensity; < 63.5, 3 = strong positive). With values obtained, the total modified H-scores were calculated. Ten fields were chosen randomly in each specimen.

### Co-Expression Analysis

In order to detect the co-expression characteristics of PD-1 and T cell subsets, serial section immunohistochemistry and analysis of the percentage of CD4, CD8, and PD-1 positive cells were carried out. Patient specimens before and 1 month and 3 months after administration of Nr-CWS were immersed into 10% formalin for 24 h at room temperature and then embedded with paraffin. Consecutive 4-μm sections were cut from each paraffin-embedded block. Adjacent consecutive sections were used to detect the expressions of CD4, CD8 and PD-1 using immunohistochemical method. Ten fields of the same position in each specimen of the three indexes were randomly selected and the percentages of CD4, CD8, and PD-1 positive cells were calculated respectively.

Then we used double label immunofluorescence histochemical method and laser scanning confocal microscopy (LSCM) to analyze the co-expression of PD-1 and CD4 or CD8 in patient specimens before and 1 month and 3 months after administration of Nr-CWS. The 4-µm paraffin-embedded sections were subjected to the following treatments: Gradual alcohol dewaxing, PBS rinsing, incubation in 3% H_2_O_2_, PBS rinsing, antigen repair in 0.01 Mol/L citrate buffer and PBS rinsing. Sections were incubated with two primary antibodies of anti-PD-1 (1 : 200; Abcam) and anti-CD4 (1 : 200; Abways Technology) or anti-CD8 (1 : 200; Abways Technology). Subsequently, sections were incubated with secondary antibodies of goat anti-mouse IgG Alexa Fluor 594 (1 : 200; Abways Technology) and goat anti-rabbit IgG Alexa Fluor 488 (1 : 200; Abways Technology) and mounted. LSCM was performed on an Olympus FV1200MPE LSCM. For the results of double label staining, the PD-1^+^CD4^+^ cells/CD4^+^ cells ratio and PD-1^+^CD8^+^ cells/CD8^+^ cells ratio in the cervical epithelium and stroma were calculated.

### Total RNA Isolation and qRT-PCR

To analyze the mRNA levels of cytokines including interleukin-10 (IL-10), interleukin-12 (IL-12), interferon-γ (IFN-γ) and tumor necrosis factor-α (TNF-α), total RNAs were extracted from cervical tissue samples using TRIzol and reverse transcribed with a PrimeScript® RT reagent Kit (TaKaRa Biotechnology, Dalian, China) for qRT-PCR using SYBR® Premix Ex Taq™ (TaKaRa Biotechnology) following the manufacturer’s instructions. The purity and amount of RNA were determined by measuring the OD260/280 nm ratio. The mRNA levels were normalized using β-actin as the housekeeping gene and analyzed by the 2^-^δδ^Ct^ method. The primers were provided by Invitrogen (Carlsbad, CA, USA) (**Table 1**).

**Table 1:** Forward and reverse primers of cytokines and their oligo sequences.

Primer name	Oligo sequence (5′ to 3′)
IL-12 forward	AGAACTTGCAGCTGAAGCC
IL-12 reverse	CCTGGACCTGAACGCAGAAT
IFN-γ forward	CTTGAATGTCCAACGCAAAGC
IFN-γ reverse	TCTTCGACCTCGAAACAGCAT
TNF-α forward	GCTGCACTTTGGAGTGATCG
TNF-α reverse	TCACTCGGGGTTCGAGAAGA
IL-10 forward	AGGCAACCTGCCTAACATGC
IL-10 reverse	GTTCTCAGCTTGGGGCATCA
β-actin forward	CGCCACCAGTTCGCCATGGA
β-actin reverse	TACAGCCCGGGGAGCATCGT

### Statistical Analysis

Data are presented as means ± SDs. Statistical analysis was performed with SPSS version 19.0 (SPSS, Inc., Chicago, IL, USA). Statistical significance was determined by independent samples T-test or one-way Analysis of Variance (ANOVA). A *P* value less than 0.05 was considered to be significant.

## Results

### Subject Characteristics of the Study Groups

The subject characteristics of the study groups are summarized in **Table 2**. The 23 recruited patients for Nr-CWS treatment were aged between 22 and 56 years old (mean 37.39 ± 9.15 years old). In these 23 patients, a single HR-HPV type was detected in 11 (47.83%, 11/23), and two or more HR-HPV types were found in the same sample from 12 (52.17%, 12/23) women. The 5 patients in the normal control group were aged between 32 and 39 years old (mean 35.6 ± 2.61 years old), and no significant differences were found compared with the Nr-CWS treatment group. No HR-HPV infection was detected in these normal control patients.

**Table 2:** Subject characteristics of the study groups.

	Nr-CWS group (n=23)			normal control group (n=5)
	Before Nr-CWS	1-Month	3-Month	
		After Nr-CWS	After Nr-CWS	
Age range (years)	22–56			32–39
Mean age (years)^1^	37.39 ± 9.15			35.6 ± 2.61
Subjects infected with HR-HPV type				
Single infection	47.83% (11/23)	–	26.09% (6/23)	0
Multiple infection	52.17% (12/23)	–	13.04% (3/23)	0
HR-HPV overcast rate	–	–	60.87% (14/23)	–
Pathological features				
CIN I	20	–	2	–
CIN II	3	–	0	–
Effective rate in pathological changes	–	–	91.3% (21/23)	–

CIN, cervical intraepithelial neoplasia; HR-HPV, high-risk human papillomavirus.

^1^mean ± SD.

At 3 months after Nr-CWS treatment, the results of HR-HPV detection in 14 out of the 23 cases became negative, and HR-HPV overcast rate was 60.87% (14/23). Besides, HR-HPV types were reduced in 3 other patients with multiple HR-HPV infections. Pathological efficient changes were observed in 21 out of the 23 cases, and the effective rate was 91.3% (21/23) at 3 months after Nr-CWS treatment.

### Expressions of CD3^+^ T, CD4^+^ T and CD8^+^ T Cell Detected by Immunohistochemistry

**Figure 1:**
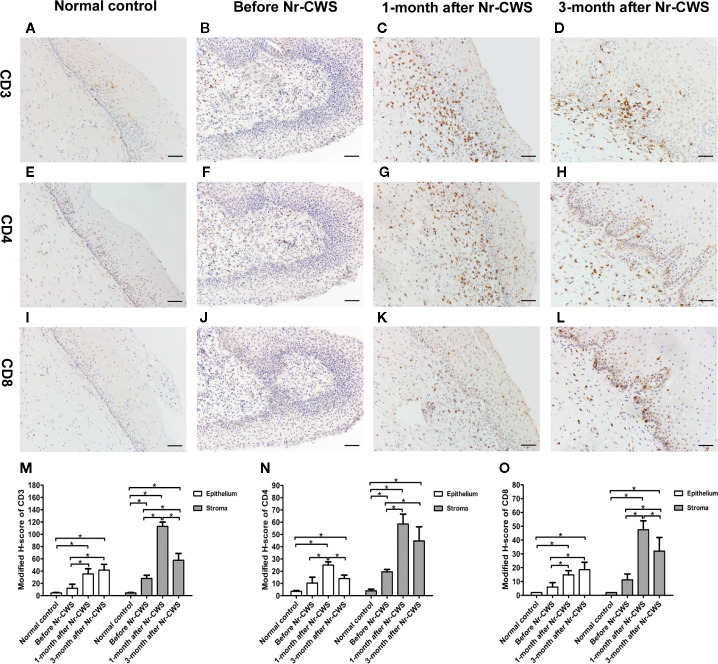
Expressions of CD3, CD4, and CD8 detected by immunohistochemistry. The expressions of CD3 were detected in cervical tissues of the normal control group **(A)**, recruited patients before Nr-CWS treatment **(B)**, 1 month after Nr-CWS **(C)**, and 3 months after Nr-CWS **(D)**. Statistical data of CD3 **(M**: ^*^ *P* < 0.05). The expressions of CD4 were detected in the normal control group **(E)**, recruited patients before Nr-CWS treatment **(F)**, 1 month after Nr-CWS **(G)**, and 3 months after Nr-CWS **(H)**. Statistical data of CD4 **(N**: ^*^ *P* < 0.05). The expressions of CD8 were detected in the normal control group **(I)**, recruited patients before Nr-CWS treatment **(J)**, 1 month after Nr-CWS **(K)** and 3 months after Nr-CWS **(L)**. Statistical data of CD8 (**O**: ^*^ *P* < 0.05). (**A–L**, scale bars = 50 μm).

The CD3^+^ T, CD4^+^ T, or CD8^+^ T cells were found both in human cervical squamous epithelium and in the underlying stroma in each group (**Figures 1A–L**). The brownish-yellowish deposits were distributed in the cytoplasm and on the cell membrane of the positive cells. The positive cells in human cervical epithelium were mainly located in the basal or intermediate layers and rarely appeared in the superficial layers.

**Figures 1M–O** showed that in the human cervical epithelial tissues of these recruited patients at 1 month after Nr-CWS administration, the modified H-scores of CD3^+^ T, CD4^+^ T, and CD8^+^ T cells were significantly higher than those before Nr-CWS treatment (*P* < 0.05). At 3 months after Nr-CWS treatment, the CD3^+^ T and CD8^+^ T cells expression scores continued to increase and reached the highest level (*P* < 0.05). The CD4^+^ T cells scores peaked at 1 month and dropped at 3 months. In the stroma underlying human cervical epithelium, the scores of CD3^+^ T, CD4^+^ T, and CD8^+^ T cells were significantly higher at 1 month than those before Nr-CWS (*P* < 0.05) and reached the highest level. At 3 months, CD3^+^ T, CD4^+^ T, and CD8^+^ T cells expression scores dropped, but were still significantly higher than those before treatment (*P* < 0.05).

### Expressions of PD-1 and PD-L1 Detected by Immunohistochemistry

**Figure 2:**
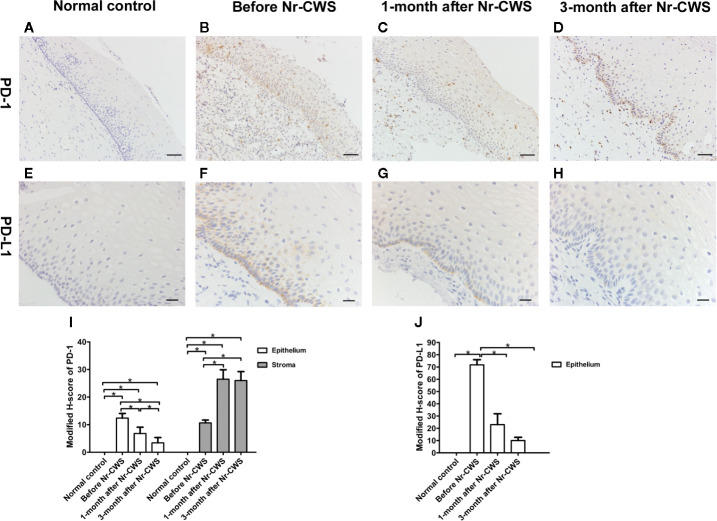
Expressions of PD-1 and PD-L1 detected by immunohistochemistry. The expressions of PD-1 were detected in cervical tissues of the normal control group **(A)**, recruited patients before Nr-CWS treatment **(B)**, 1 month after Nr-CWS **(C)** and 3 months after Nr-CWS **(D)**. Statistical data of PD-1 (**I**: ^*^ *P* < 0.05). The expressions of PD-L1 were detected in the normal control group **(E)**, recruited patients before Nr-CWS treatment **(F)**, 1 month after Nr-CWS **(G)** and 3 months after Nr-CWS **(H)**. Statistical data of PD-L1 (**J**: ^*^ *P* < 0.05). (**A–D**, scale bars = 50 μm; **E–H**, scale bars = 20 μm).

**Figures 2A–D, I** showed the PD-1 expression in normal control and recruited patients. The PD-1^+^ cells with brownish-yellowish deposits in cytoplasm and cell membrane were distributed in human cervical epithelium and stroma. In the normal control group, PD-1 expression was almost absent. Before these patients were treated with Nr-CWS, the expression scores of PD-1 in human cervical epithelium and stroma were significantly higher than those of normal controls (*P* < 0.05). After Nr-CWS treatment, the PD-1 expression in the human cervical epithelial tissue decreased gradually with the extension of repair time, which was statistically significant compared with that before Nr-CWS treatment (*P* < 0.05). However, in human cervical stroma, the expression of PD-1 increased after Nr-CWS treatment (*P* < 0.05), which might be due to increased lymphocyte infiltration.

**Figures 2E–H, J** showed the PD-L1 expression in normal control and recruited patients. No PD-L1 expression was detected in human cervical tissue of the normal control group. Before the Nr-CWS treatment of these patients, PD-L1 expression was detected in human cervical epithelial cells, mainly in the basal layer of epithelium. The brownish-yellowish deposits were located in the cytoplasm and membrane of the PD-L1 positive cells. After administration of Nr-CWS, the expression level of PD-L1 strikingly reduced (*P* < 0.05) as the repair time prolonged.

### Co-Expression Analysis of PD-1 and CD4 or CD8

**Figure 3:**
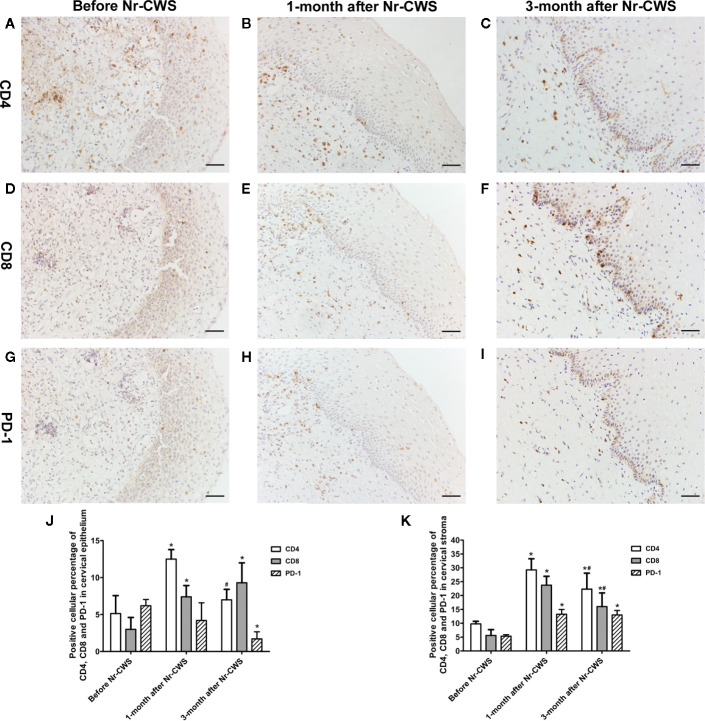
Co-expression analysis of PD-1 and CD4 or CD8 by serial section immunohistochemistry. The immunohistochemistry results of co-expression analysis revealed the expressions of CD4, CD8, and PD-1 at the same position in the serial sections. CD4 expression in recruited patients before Nr-CWS **(A)**, 1 month after Nr-CWS **(B)** and 3 months after Nr-CWS **(C)**; CD8 expression in recruited patients before Nr-CWS **(D)**, 1 month after Nr-CWS **(E)** and 3 months after Nr-CWS **(F)**; PD-1 expression in recruited patients before Nr-CWS **(G)**, 1 month after Nr-CWS **(H)** and 3 months after Nr-CWS **(I)**. Statistical data of CD4, CD8, and PD-1 in the cervical epithelial tissue (**J**: ^*^ *P* < 0.05 vs Before Nr-CWS, ^#^ *P* < 0.05 vs 1-month after Nr-CWS). Statistical data of CD4, CD8 and PD-1 in the cervical stroma (**K**: ^*^ *P* < 0.05 vs Before Nr-CWS, ^#^ *P* < 0.05 vs 1-month after Nr-CWS). (**A–I**, scale bars = 50 μm).

The immunohistochemical results of co-expression analysis revealed the expressions of CD4, CD8 and PD-1 at the same position in the serial sections of human cervical tissue (**Figures 3A–I**). **Figure 3J** showed that in the human cervical epithelial tissue, the percentage of CD4 positive cells increased significantly after Nr-CWS treatment from 5.14% to 12.5% at 1 month (*P* < 0.05) and 7% at 3 months (*P* < 0.05), and reached a peak at 1 month. The percentage of CD8 positive cells kept increasing significantly after Nr-CWS treatment from 3% to 7.4% (at 1 month) (*P* < 0.05) and 9.29% (at 3 months) (*P* < 0.05), and peaked at 3 months. However, after Nr-CWS treatment, the percentage of PD-1^+^ cells showed a continuous downward trend, from 6.2% before Nr-CWS treatment to 4.2% at 1 month and 1.71% at 3 months, and was significantly lower at 3 months than before Nr-CWS treatment (*P* < 0.05). In human cervical stroma (**Figure 3K**), the percentages of CD4^+^ T and CD8^+^ T cells increased significantly from 9.75% and 5.6% respectively after Nr-CWS treatment, reaching their respective peaks of 29.25% (*P* < 0.05) and 23.75% (*P* < 0.05) at 1 month. The percentages of CD4^+^ T and CD8^+^ T cells at 3 months were 22.33% and 16% respectively, both of which were significantly higher than those before Nr-CWS treatment (*P* < 0.05). The percentage of PD-1 positive cells was 5.33% before treatment, and significantly increased to 13.25% (at 1 month) (*P* < 0.05) and 13% (at 3 months) (*P* < 0.05) after Nr-CWS treatment.

**Figure 4:**
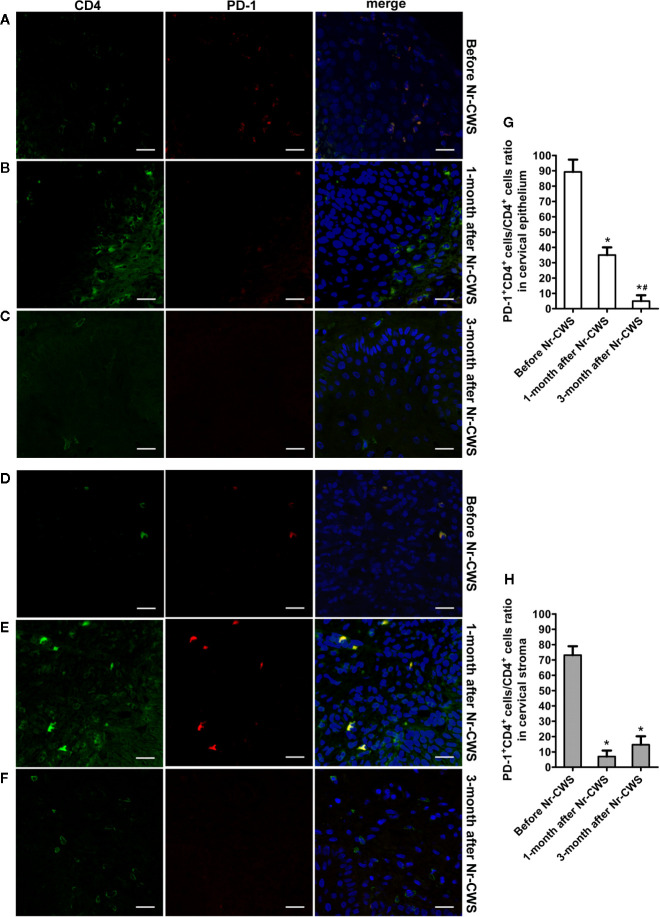
Co-expression analysis of PD-1 and CD4 by double label immunofluorescence histochemical method. The co-expression of PD-1 and CD4 was detected by double label immunofluorescence histochemical method: the co-expression of PD-1 and CD4 in human cervical epithelium of the recruited patients before Nr-CWS treatment **(A)**, 1 month after Nr-CWS **(B)** and 3 months after Nr-CWS **(C)**; the co-expression of PD-1 and CD4 in human cervical stroma of the patients before Nr-CWS treatment **(D)**, 1 month after Nr-CWS **(E)** and 3 months after Nr-CWS **(F)**; statistical data of co-expression of PD-1 and CD4 (**G, H**: ^*^ *P* < 0.05 vs Before Nr-CWS, ^#^ *P* < 0.05 vs 1-month after Nr-CWS) (**A–F**, scale bars = 20 μm).

**Figure 5:**
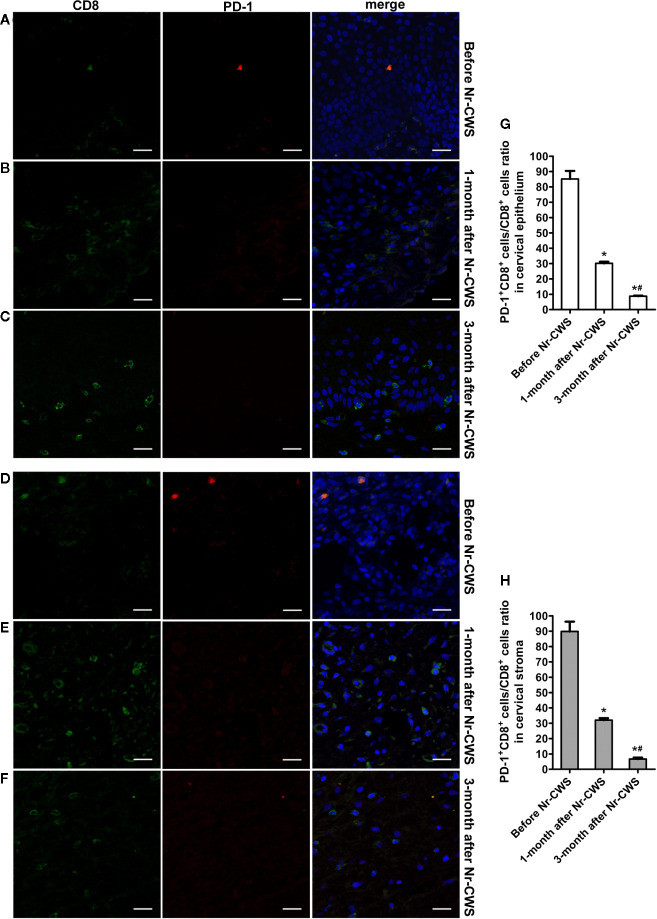
Co-expression analysis of PD-1 and CD8 by double label immunofluorescence histochemical method. The co-expression of PD-1 and CD8 was detected by double label immunofluorescence histochemical method: the co-expression of PD-1 and CD8 in human cervical epithelium of the recruited patients before Nr-CWS treatment **(A)**, 1 month after Nr-CWS **(B)** and 3 months after Nr-CWS **(C)**; the co-expression of PD-1 and CD8 in human cervical stroma of the patients before Nr-CWS treatment **(D)**, 1 month after Nr-CWS **(E)** and 3 months after Nr-CWS **(F)**; statistical data of co-expression of PD-1 and CD8 (**G, H**: ^*^ *P* < 0.05 vs Before Nr-CWS, ^#^ *P* < 0.05 vs 1-month after Nr-CWS) (**A–F**, scale bars = 20 μm).

**Figures 4** and **5** showed the results of PD-1 and CD4 (or CD8) double label immunofluorescence histochemical method. Besides PD-1^+^CD4 ^+^ (or PD-1^+^CD8^+^) cells, much more PD-1^-^CD4^+^ (or PD-1^-^CD8^+^) cells appeared in human cervical epithelium and stroma after Nr-CWS treatment. In addition, some PD-1^+^CD4^-^ (or PD-1^+^CD8^-^) cells were observed in human epithelium and stroma before treatment. The PD-1^+^CD4^+^ cells/CD4^+^ cells ratio and PD-1^+^CD8^+^ cells/CD8^+^ cells ratio in human cervical epithelium and stroma were calculated separately. After Nr-CWS treatment, the PD-1^+^CD4^+^ cells/CD4^+^ cells ratio significantly fell from 89.26% to 35% at 1 month (*P* < 0.05) and 5% at 3 month (*P* < 0.05) in the cervical epithelium, from 73.15% to 7% at 1 month (*P* < 0.05) and 14.7% at 3 month (*P* < 0.05) in the cervical stroma. The PD-1^+^CD8^+^ cells^/^CD8^+^ cells ratio significantly fell from 85.16% to 30.14% at 1 month (*P* < 0.05) and 8.74% at 3 month (*P* < 0.05) in the cervical epithelium, from 89.83% to 32.03% at 1 month (*P* < 0.05) and 6.69% at 3 month (*P* < 0.05) in the cervical stroma.

### Detection of Cytokine Levels in Human Cervical Tissue by qRT-PCR

**Figure 6:**
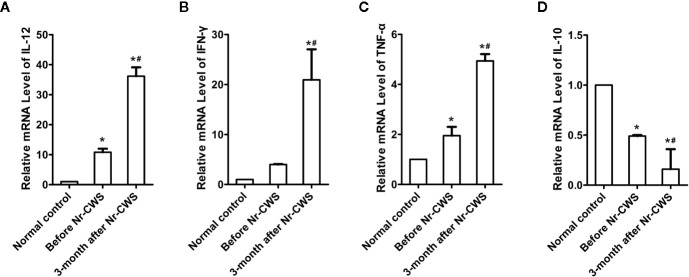
Detection of cytokine mRNA levels in cervical tissue by qRT-PCR. Statistical data of the mRNA levels of local pro-inflammatory cytokines: IL-12 **(A)**, IFN-γ **(B)**, TNF-α **(C)**; Statistical data of the mRNA level of suppressive cytokine IL-10 **(D)** (^*^ *P* < 0.05 vs Normal control, ^#^ *P* < 0.05 vs Before Nr-CWS).

The effect of Nr-CWS on cytokines in human cervical tissues was analyzed by qRT-PCR (**Figure 6**). Results showed that compared with those in the normal control, the mRNA levels of local pro-inflammatory cytokines (IL-12, IFN-γ, and TNF-α) were higher and the mRNA level of suppressive cytokine IL-10 was lower in the cervical tissues of these patients before Nr-CWS treatment. At 3 months after Nr-CWS, the mRNA levels of IL-12, IFN-γ, and TNF-α elevated significantly (*P* < 0.05) while the level of IL-10 decreased significantly (*P* < 0.05).

## Discussion

Over 100 subtypes of HPV exist with 15 being identified as high risk. Persistence of HR-HPV infection in tissues is critical factor in the development of CIN and ultimately, carcinoma. Therefore, in this study, patients with HR-HPV infection and CIN were selected as research objects to detect the therapeutic effect of Nr-CWS on cervical cancer-related precancerous lesions, so as to prevent the occurrence of human cervical cancer. Nevertheless, the standard-of-care management of CIN 2 and CIN 3 is mainly surgical therapy which ablates or excises in human cervical transformation zone (17). So in this study, the subjects recruited in the actual clinical practice were mainly CIN 1 and few CIN 2 or higher patients.

The surgical therapy carries potential risks such as preterm birth (17). For patients with fertility requirements, there has been a long-standing and continued interest in the development of a safe, effective topical therapy. In the present study, Nr-CWS was applied locally in cervical tissues of patients with HR-HPV infection and CIN. Tissue samples from recruited patients were collected at 1 month after the application of Nr-CWS to detect short-term changes in cervical local immunity, and cervical tissue samples were collected at 3 months after Nr-CWS to detect local immune changes after repair for a period of time. For some reasons of clinical application, the placebo group was not set in this study, but the cervical local immune status of the patients before Nr-CWS treatment was compared with that of the normal control group. The former reflected the immune status in the absence of drug treatment for HR-HPV infection. The results showed that the HR-HPV overcast rate was about 60.87% after 3 months of repair, which was higher than the natural HPV negative conversion rate (26.9%) within 3 months (18). Meanwhile, the effective rate of pathological changes by removing dysplastic cells was 91.3% at 3 months after Nr-CWS treatment. The results showed that clinical application of Nr-CWS had a good clinical effect and may eliminate the HR-HPV infection and dysplastic cells in cervix of a considerable proportion of patients.

Cell-mediated immunity of an individual is considered to be a vital mechanism in protection against the virus and elimination of virus-infected cells. The activation of CD4^+^ T cells and consequently synthesis of cytokines are essential in the immune response because these mediators could activate or inhibit other cells types including cytotoxic CD8^+^ T cells (19). Cytotoxic CD8^+^ T cell infiltrates appear to be principal effectors in eliminating HPV infected pre-neoplastic human cervical epithelial cells and severe dysplastic cells (20). An increasing number of documents have described the relationship between CIN and T cell subsets. The women presenting CIN had a higher CD4^+^ T and CD8^+^ T cell infiltrates than the healthy ones (21), which was consistent with our results. The presence of CD4^+^ T cells in the stroma of women with CIN 2/3 was predictor for the regression of lesions (22). The patients with regressed CIN 2/3 had higher CD8^+^ T cells than patients that presented persistent lesions (23). Low CD3^+^ T and CD8^+^ T cells infiltrates were reported as being predictive markers of progressive cervical disease (24). In this study, after administration of Nr-CWS treatment, the infiltrates of CD3^+^ T, CD4^+^ T, and CD8^+^ T cells improved significantly both in human cervical epithelium and stroma, suggesting an improvement in local cell immunity by Nr-CWS. Additionally, T cells infiltrate from human cervical stroma to the epithelium, which is matched by our results. We found the positive cells in epithelium were mainly located in the basal or intermediate layers and rarely appeared in the superficial layers. Under the stimulation of Nr-CWS, T cells in human cervical stroma changed more rapidly than those in human cervical epithelium, and the increase was higher than that in human cervical epithelium. Furthermore, cytotoxic CD8^+^ T cells are orchestrated by CD4^+^ T cell responses. The activation of CD4^+^ T cells occurs earlier, with the production of cytokines that are essential to the recruitment and activation of CD8^+^ T cells. In line with this, we found that the CD4^+^ T cells in the epithelium reached the highest level at 1 month after Nr-CWS, while the CD8^+^ T cells gradually increased and peaked at 3 months after Nr-CWS. In addition, the results showed that the scores of CD3^+^ T, CD4^+^ T and CD8^+^ T cells in the stroma increased significantly at 1 month, indicating a strong local immune response, while the decrease in the scores of immune cells at 3 months indicated a decrease in the intensity of the immune response. This may be related to the reduction of HPV, the improvement of the condition, and therefore the corresponding weakening of the immune response. The changes of T cells in the cervical epithelium were slower than those in the stroma, and only CD4^+^ T cells showed a similar trend, while the CD8^+^ T cells showed a later change than CD4^+^ T cells, reaching a peak at 3 months.

Inhibitory receptors are crucial negative regulatory pathways, controlling autoreactivity and immunopathology. Although inhibitory receptors are transiently expressed in functional effector T cells during activation, their sustained high expression are hallmarks of exhausted T cells (25). PD-1 is illustrated as an important negative costimulatory molecule. The binding of PD-1 and PD-L1 can block TCR and its co-stimulus signal transduction, inhibit the activation and proliferation of T cells, deplete the function of effector T cells. The PD-1/PD-L1 pathway has been revealed to inhibit a wide range of immune responses against pathogens, tumors and self-antigens (26).

PD-L1 is widely expressed in activated T and B cells, antigen presenting cells and thymic cortical epithelial cells (27). Relevant studies have found abnormal PD-L1 expression in various cancers including cervical squamous cell carcinoma (CSCC). Mezache et al. reported that PD-L1 was a solid biomarker of productive HPV infection in cervix and was significantly upregulated in both cancer cells and surrounding inflammatory cells in human cervical cancer (28). As for CIN, Yang et al. proved that increase in PD-L1 and PD-1 expression negatively regulated cervical cell immunity to HPV, and contributed to the progression of HR-HPV related CIN (29). The expressions of PD-1 and PD-L1 in CIN and CSCC were of prognostic value and associated with HPV status (30). In the study of Mezache et al (28), the percentage of cases with high PD-L1 expression in the abnormal squamous cells of CIN 1-2 lesions was 95%. PD-L1 positive mononuclear cells, which were abundant in the invasive squamous cell nests of CSCC, were rarely evident in the epithelial layer of CIN 1-2. Consistent with that, because the participants were mainly CIN 1 or CIN 2 patients, our results showed that PD-L1 was expressed in the cervical epithelial cells. However, PD-L1 expression was not detected in other cells such as mononuclear cells, which may be related to the relatively mild lesion level of the recruited patients. In addition, similar to previous findings, the PD-L1 expression was located mainly toward the basal layer of epithelium, where E6 and E7 RNAs might be relatively abundant (28). After treatment with Nr-CWS, the expression of PD-L1 decreased significantly, demonstrating that Nr-CWS had a certain inhibitory effect on the PD-1/PD-L1 pathway.

PD-1 is expressed on activated T cells, B cells and some myeloid cells, though its functions are best characterized for T cells. PD-1 interacts with its ligands, PD-L1 and PD-L2. There is accumulating evidence indicating the relationship between PD-1 expression and impaired cellular immunity. The sustained upregulation of PD-1 in CD4^+^ T cells and CD8^+^ T cells was one of the characteristic features of T cell exhaustion during chronic infection or cancer (25, 31). As for HPV infection and CIN, PD-1 expression on cervical T cells was associated with HR-HPV positivity and increased in parallel with increasing CIN grade (29). The PD-1 expression levels on peripheral CD4^+^ T and CD8^+^ T cells were significantly elevated in samples from patients with cancer and CIN (26). As for the function of exhausted T cells, the exhausted CD8^+^ T cells lose effector functions including production of IL-2, IFN-γ, TNF-α and β-chemokines, high proliferative capacity, ex-vivo cytolytic activity and degranulation (32). The exhaustion of CD4^+^ T cells shares many characteristics with CD8^+^ T cell exhaustion, including impaired production of effector cytokines (e.g. IFN-γ, TNF-α) (33). On the other hand, soluble molecules are also important signals in regulating T cell exhaustion, including suppressive cytokines such as IL-10 and transforming growth factor-β (TGF-β) and pro-inflammatory cytokines such as type I IFN and IL-6 (25).

The present study first demonstrated that Nr-CWS treatment remarkably raised the percentages of CD4^+^ T and CD8^+^ T cells in both the cervical epithelium and the stroma of the patients, and the percentage of PD-1^+^ cells decreased in human cervical epithelium. However, the percentage of PD-1^+^ cells in human cervical stroma increased after Nr-CWS treatment. In order to find out the exact effect of Nr-CWS on PD-1 and T cells, we designed a co-expression analysis experiment. The results showed that PD-1^+^CD4^+^ cells/CD4^+^ T cells ratio and PD-1^+^CD8^+^ cells/CD8^+^ T cells ratio decreased both in cervical epithelium and stroma, suggesting that Nr-CWS treatment reduced local exhausted CD4^+^ T and CD8^+^ T cells and more uninhibited CD4^+^ T and CD8^+^ T cells were present in human cervix. The increase in the percentage of PD-1^+^ cells in the stroma after Nr-CWS treatment may be caused by the significant increase in the percentages of CD4^+^ T and CD8^+^ T cells. Additionally, PD-1 is also expressed on other immune cells besides CD4^+^ T and CD8^+^ T cells. We found some PD-1^+^CD4^-^ (or PD-1^+^CD8^-^) cells in the image of double label staining, which may be other cells expressing PD-1 except CD4^+^ T or CD8^+^ T cells. On the other hand, the mRNA levels of local pro-inflammatory cytokines (IL-12, IFN-γ, and TNF-α) were elevated while the mRNA level of suppressive cytokine IL-10 decreased after Nr-CWS treatment, which also verified the efficient role of Nr-CWS in reversal T cell exhaustion. The role of Nr-CWS in regulating cytokines may also be caused by affecting other immune cells. In short, the mechanisms of Nr-CWS in subverting T cell exhaustion and revitalizing host immunity are complex. Much more researches are needed to clarify the immune enhancing effect of Nr-CWS.

## Conclusion

In light of these results, we propose that Nr-CWS, as an immunotherapeutic agent for HR-HPV infection and CIN, plays an efficient part in immune enhancement and treatment, through upregulating T cell subsets and inhibiting PD-1/PD-L1 signal pathway.

## Data Availability Statement

The original contributions presented in the study are included in the article/supplementary materials. Further inquiries can be directed to the corresponding authors.

## Ethics Statement

The studies involving human participants were reviewed and approved by the ethics committee of the First Hospital of Hebei Medical University and the ethics committee of the Fourth Hospital of Hebei Medical University. The patients/participants provided their written informed consent to participate in this study.

## Author Contributions

LZ and JT contributed to the conception and design of the study. WC designed experiments, analyzed the samples, and contributed to the manuscript preparation. YiZ contributed to the design of the experiments and study supervision. CZ performed experiments. SS contributed to the project administration. XL and XB were responsible for clinical patient recruitment and treatment. YaZ, QG and QL collected samples from the patients and performed experiments. All authors contributed to the article and approved the submitted version.

## Funding

This work was supported by Technology Development Agreement of Hebei Medical University and Weihai Greatest Pharmaceutical Research Institute Co., Ltd., RP China, and Nature Science Foundation of Hebei Province [Grant No. H2017206078]. The funder bodies were not involved in the study design, collection, analysis, interpretation of data, the writing of this article or the decision to submit it for publication.

## Conflict of Interest

YiZ was employed by the company Weihai Greatest Pharmaceutical Research Institute Co., Ltd.

The remaining authors declare that the research was conducted in the absence of any commercial or financial relationships that could be construed as a potential conflict of interest.
